# Vaginal Mesh Exposure Following Pessary Use After Transvaginal Mesh Surgery: A Case Report

**DOI:** 10.1002/iju5.70135

**Published:** 2026-01-07

**Authors:** Yukiko Tsunoda, Kumiko Kato, Hidemori Araki, Masashi Kato, Masahiro Narushima

**Affiliations:** ^1^ Department of Female Urology Japanese Red Cross Aichi Medical Center Nagoya Daiichi Hospital Nagoya Japan; ^2^ Department of Female Urology Meitetsu Hospital Nagoya Japan; ^3^ Department of Urology Meitetsu Hospital Nagoya Japan; ^4^ Department of Urology Japanese Red Cross Aichi Medical Center Nagoya Daiichi Hospital Nagoya Japan

**Keywords:** mesh complication, mesh exposure, pelvic organ prolapse, pessary complication, transvaginal mesh surgery (TVM)

## Abstract

**Introduction:**

Vaginal pessaries are a widely used treatment for pelvic organ prolapse. They are considered minimally invasive, effective, and easy. However, pessary‐related adverse events are sometimes underestimated. Here, we report on an important case of vaginal mesh exposure as a pessary complication.

**Case Presentation:**

A patient underwent transvaginal mesh surgery for pelvic organ prolapse, and after 10 years, had a mild recurrence. Gynecologists recommended that she use a pessary. After 3 years, she had vaginal bleeding and malodor. Gynecologists detected mesh exposure, but they downplayed the finding and continued pessary use. Two years after that, she came to our hospital and underwent mesh excision surgery. She had an uneventful postoperative course with no complications in the 2 years after the surgery.

**Conclusion:**

We propose that pessary use after mesh surgery can cause mesh exposure, which must not be ignored because it may lead to mesh infection and intra‐abdominal abscess.

**Keynote Message**

Vaginal pessaries are a simple and effective treatment for POP. However, serious complications such as vesicovaginal and rectovaginal fistulas have been reported. We present a case of vaginal mesh exposure that occurred 13 years after TVM, after 3 years of pessary use.

**Abbreviations**
LSC: laparoscopic sacrocolpopexy
POP: pelvic organ prolapse
TVM: transvaginal mesh prolapse surgery

## 1 Introduction

Vaginal pessaries are considered a noninvasive, practical treatment for pelvic organ prolapse (POP) [1]. However, they are associated with complications including vaginal infections and erosions (pressure ulcers) leading to bleeding, discharge, and malodor [1]. Although rare, more serious complications must not be forgotten. These include vaginal impaction, vesicovaginal and rectovaginal fistula, and vaginal vault perforation with bowel evisceration [2, 3].

Vaginal mesh exposure is a well‐known complication after POP surgeries using mesh such as transvaginal mesh surgery (TVM) and laparoscopic sacrocolpopexy (LSC). In contrast, complications caused by pessary use after POP have been overlooked by researchers. Therefore, we present a case of vaginal mesh exposure following 3 years of pessary use, 13 years after TVM surgery.

## 2 Case Presentation

**FIGURE 1:**
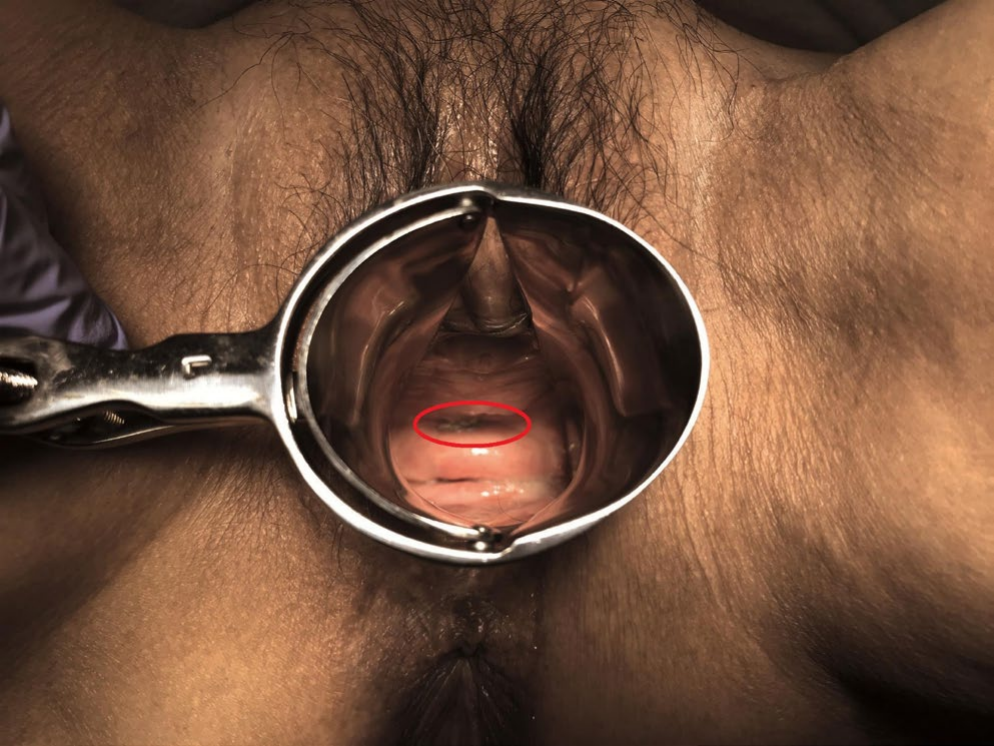
Vaginoscopic findings (cystoscope was used). There was an 8 by 10 mm mesh exposure in the patient's anterior vaginal wall, and the mesh had stones.

**FIGURE 2:**
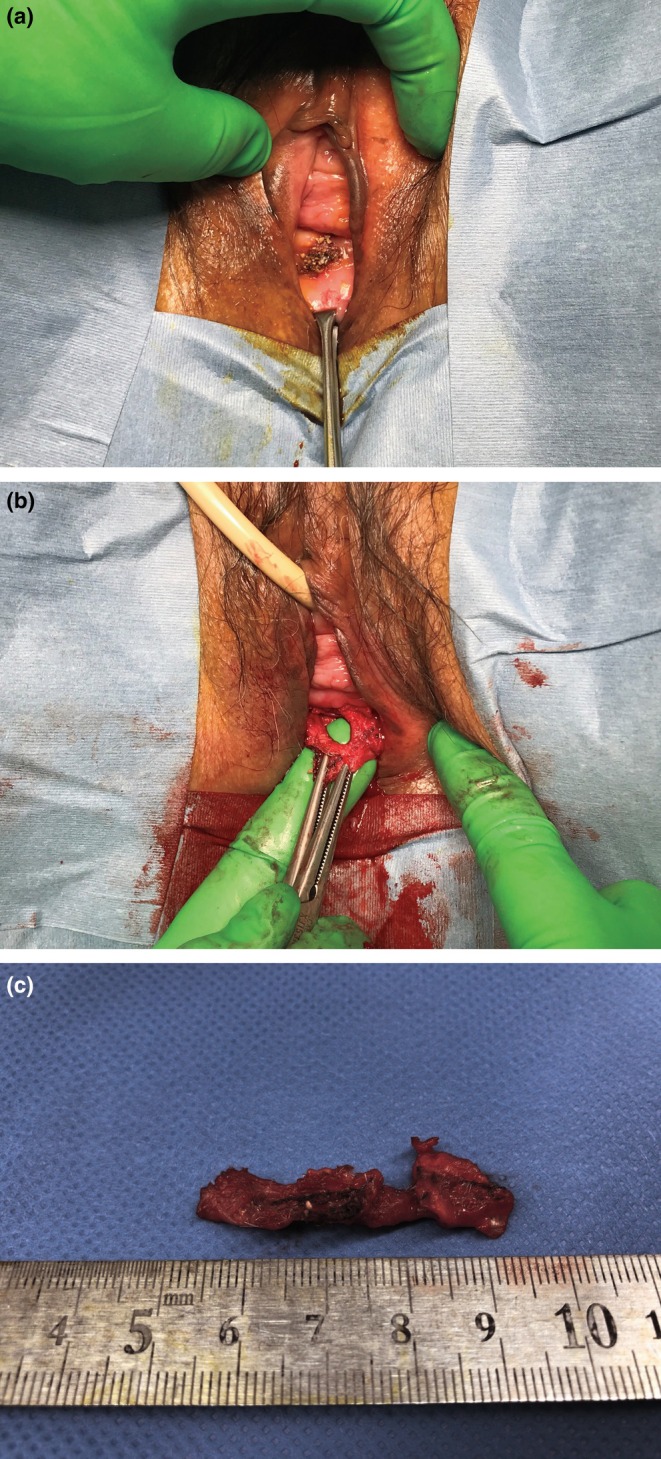
Surgical findings. (a) Exposed mesh under spinal anesthesia. (b) After dissecting the exposed mesh from the patient's anterior vaginal wall. (c) The excised mesh.

At the age of 53, the patient underwent anterior TVM surgery to treat POP in the Department of Gynecology at a general hospital. Polypropylene mesh (Gynemesh) was attached between the urinary bladder and the anterior vaginal wall. At age 63, she experienced a descending sensation in her vagina and detected an egg‐sized mass with her fingers while bathing. She revisited the general hospital, but the operating surgeon had retired, and the attending physician had limited experience in urogynecology. She was diagnosed as having POP recurrence, and vaginal pessaries were recommended. She then continued to visit the hospital every 6 months. After 3 years of pessary use, she began to experience vaginal bleeding and malodor. A gynecologist diagnosed the development of vaginal mesh exposure but advised her to continue pessary use. At age 67, she was referred to a urology clinic for pessary follow‐up, and the urologist, in turn, referred her to our hospital. During her vaginal examination, we removed her pessary and confirmed an 8 by 10 mm mesh exposure in her anterior vaginal wall, where the pessary made contact. The complication was coded as 2B/T4/S2 according to the IUGA/ICS classification system (Figure 1). She had stage II cystocele. POP‐Q score was Aa+1, Ba+1, C‐4, gh3, pb2.5, tvl7.5, Ap‐2.5, Bp‐2.5, and D‐7. We explained the possibility of bleeding and odor worsening, and she agreed to undergo partial mesh excision under spinal anesthesia. After water dissection, the exposed part of the mesh was pulled with a clamp, and as much mesh as possible was dissected and excised (Figure 2). The excised mesh was 4 cm long with contracture deformity. We sutured her anterior vaginal wall vertically. Her postoperative course was uneventful. She underwent pelvic floor muscle training, and her POP‐Q score 2 years after surgery was: Aa‐1.5, Ba‐1.5, C‐6, gh2.5, pb3, tvl7, Ap‐3, Bp‐3, and D‐7. There was no bleeding, vaginal discharge, malodor, nor recurrence of mesh exposure in 2 years of follow‐up. Also, she felt no POP symptoms.

## 3 Discussion

In this case, when POP reoccurred 10 years after TVM surgery, a pessary was inserted. Pessaries seem to offer an easy solution to POP. However, this raises the concern that pessaries may be used for patients with mild symptoms and may not always be necessary. In this case, too, there was no more than a 2‐cm descent below the hymen ring which could be well managed by pelvic floor muscle training. Complication rates from pessary use vary in reports from at least 11% to at most 73% [1]. Therefore, the risk of complications should be considered more carefully.

A review of 58 studies showed that risk factors for mesh exposure after POP surgeries are: smoking, diabetes mellitus, patient age, surgery‐related factors, postoperative urethral dilatation, excessive sling tensioning, surgeon experience, combined vaginal and abdominal approach for mesh placement, inverted T colpotomy, and concomitant hysterectomy [4]. Systematic reviews of TVM showed that the rate of vaginal exposure often reached > 10% in Western countries [5], whereas Japanese studies reported the rate was 0.7%–3.2% [6, 7, 8, 9, 10, 11]. Several factors might contribute to this, such as dissection at the appropriate layer, avoidance of concomitant hysterectomy and midurethral sling [9, 10], and lower sexual activity among Japanese older adults [9]. The LSC incidence of vaginal exposure is reported to be 0%–5% except for high incidence (23%) of LSC combined with total vaginal hysterectomy [12, 13].

In this case, it was predictable that the patient's mesh could become exposed by these two facts: vaginal mesh exposure is one of the major complications of POP surgeries, and pessary use can cause ischemia and vaginal erosion. Mesh exposure can cause mesh infection, then intraperitoneal infection. Immediate action should have been taken at the time mesh exposure was first noticed. Local estrogen is considered an effective treatment for asymptomatic, minor mesh exposure. However, in this case, since the patient had vaginal bleeding and malodor, we chose to proceed with mesh excision.

In the UK Clinical Guideline for the use of vaginal pessaries, it is stated that a pessary should not be considered when there is identifiable synthetic vaginal mesh erosion, and that a pessary may be an option but additional caution is required when synthetic mesh has been placed in the vagina during previous surgery and/or when there is preexisting vaginal pain [14].

The Joint writing group of the American Urogynecologic Society and the Society of Urologic Nurses and Associates also established a clinical consensus statement on pessary use. They said that there are limited data regarding pessary use in women with vaginal mesh erosion, severe postirradiation scarring, nonhealing ulcers, undiagnosed vaginal bleeding, and severe vaginal infections. Furthermore, reviewing the results from the Delphi survey, they agreed that there is likely a higher rate of pessary complications, so clinicians should avoid using pessaries in women with these conditions [15]. If pessaries must be used in patients who have undergone TVM surgery, they should be informed of the risk of mesh exposure, and the need for frequent follow‐up.

## 4 Conclusion

We found a case of a pessary‐derived mesh exposure which may have led to a missed opportunity for early treatment. Although there are limited data indicating that a pessary is a risk factor for mesh exposure, many experienced urogynecologists recognize the risk of using a pessary after mesh surgery.

Therefore, clinicians have to be aware of the risk of mesh exposure in cases of pessary use after mesh surgeries. Pessary therapy in such cases should only be undertaken with heightened caution and under close clinical surveillance.

## Consent

The patient gave written consent.

## Conflicts of Interest

The authors declare no conflicts of interest.

## Data Availability

Data sharing not applicable to this article as no datasets were generated or analyzed during the current study.
